# Synergizing Conformal Lithiophilic Granule and Dealloyed Porous Skeleton toward Pragmatic Li Metal Anodes

**DOI:** 10.1002/smsc.202100110

**Published:** 2022-02-06

**Authors:** Zixiong Shi, Zhongti Sun, Xianzhong Yang, Chen Lu, Shuo Li, Xiaoyu Yu, Yifan Ding, Ting Huang, Jingyu Sun

**Affiliations:** ^1^ College of Energy Soochow Institute for Energy and Materials InnovationS (SIEMIS) Key Laboratory of Advanced Carbon Materials and Wearable Energy Technologies of Jiangsu Province Soochow University Suzhou 215006 P. R. China; ^2^ College of Materials Science and Engineering Jiangsu University Zhenjiang 212013 P. R. China

**Keywords:** dealloyed Cu skeleton, high Li utilization, Li metal batteries, lithiophilic CuSe granules, optimized Li nucleation

## Abstract

Li metal is regarded as one of the most promising anodes for next‐generation rechargeable batteries. Nonetheless, infinite volume change and severe dendrite growth impede its practicability. To date, unremitting efforts have been devoted to stabilizing Li metal anode via the rational design of 3D current collectors. In this sense, optimizing Li nucleation behavior plays a pivotal role in alleviating the dendrite formation. Herein, a practically viable route is devised by in situ crafting lithiophilic CuSe granules on the dealloyed Cu skeleton (D‐Cu@CuSe). Persuasive electrochemical analysis and systematic theoretical calculation disclose the underlying Li nucleation mechanism on the CuSe overlayer. Impressively, the D‐Cu@CuSe‐Li symmetric cell can sustain a stable plating/stripping operation over 1000 h at a high depth of discharge at 62.5%. More crucially, when paired with high‐loading sulfur cathodes, D‐Cu@CuSe‐Li||S batteries harvest advanced areal capacity and stable cycling performance even under stringent working conditions of low negative‐to‐positive (N/P) (≈2) and electrolyte‐to‐sulfur (8 μL mg_s_^−1^) ratios. Overall, a fresh perspective into rationalizing current collector design is afforded, which extends Li utilization and cycling durability in the pursuit of pragmatic Li metal anodes.

## Graphical abstract

The 3D D‐Cu@CuSe host with abundant lithiophilic granules enables optimized Li nucleation behavior and dendrite‐free Li plating morphology, accordingly extending the discharge depth and cycling lifetime of the Li metal anode for pursuing high‐energy Li metal batteries.

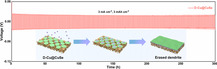

## 1 Introduction

Lithium (Li) metal anode is featured by remarkable specific capacity (3860 mAh g^−1^), low gravimetric density (0.53  g cm^−3^), and low electrochemical potential (−3.04 V versus standard hydrogen electrode), making it an appealing candidate for next‐generation rechargeable batteries with advanced energy density.^[^ ^1^, ^2^, ^3^, ^4^, ^5^, ^6^ ^]^ Nonetheless, its practical application is plagued by a myriad of intractable issues, mainly pertaining to infinite volume change and uncontrollable dendrite growth during electrochemical cycling, which will inevitably give rise to rapid capacity decay accompanied by potential safety hazards.^[^ ^7^, ^8^, ^9^, ^10^, ^11^, ^12^, ^13^ ^]^ To this end, exhaustive strategies aiming to optimize and stabilize the Li anode have been developed with an emphasis on Li host design.^[^ ^14^, ^15^, ^16^ ^]^

3D current collectors, which are conducive to cushioning volume expansion and homogenizing electric field distribution, have spurred burgeoning research interest in ameliorating Li plating/stripping operation.^[^ ^17^, ^18^, ^19^, ^20^, ^21^, ^22^, ^23^ ^]^ In this respect, it still remains a formidable challenge to acquire low Li nucleation overpotential due to the lithiophobic property of common current collectors (e.g., Cu, Ni, C), thereby inescapably triggering Li dendrite formation.^[^ ^24^ ^]^ In addition, the spontaneously derived solid electrolyte interface (SEI) is prone to crack and form repetitively, therefore further deteriorating the Li dendrite issue. Crafting extra lithiophilic matrix is of pivotal significance to guide initial plating and subsequent growth of Li.^[^ ^25^, ^26^, ^27^, ^28^ ^]^ Note that general strategies for fabricating lithiophilic layers are complicated and tend to undermine the entire energy density of Li metal batteries (LMBs), which are detrimental to their scalable production and ultimate commercialization. In addition, the pathway of Li‐ion migration on various lithiophilic candidates still lacks of comprehensive investigations, where the Li nucleation mechanism remains rather elusive.

Another major bottleneck circumventing the practicability of Li metal anode lies in its conspicuous capacity mismatch with the paired cathodes, which would lead to the shallow depth of discharge (DOD) and undesirable utilization ratio of Li anode.^[^ ^29^, ^30^ ^]^ It is worth noting that the experimental Li foil (500 μm thickness) contributes to a considerable capacity of 100 mAh cm^−2^, while the paired cathode tends to afford a far less capacity of ≈1 mAh cm^−2^. Moreover, excessive Li metal will offset the irreversible loss of active Li and weaken the detrimental effect of “dead Li”: thus‐derived longevous lifetime can hardly reflect the real stability of Li metal anode. Constructing a 3D lithiophilic Li host and dictating Li metal accommodation are therefore of vital importance, which is envisaged to enable a reasonable evaluation on the durability of Li metal anode during the repeated plating/stripping process. In further contexts, when paired with a high‐capacity cathode, the assembled LMBs are expected to harvest advanced energy density owing to the high utilization of Li metal.^[^ ^31^, ^32^ ^]^ In this sense, it is imperative and meaningful to lower the negative‐to‐positive (N/P) ratio to <5 in the pursuit of LMB commercialization, whereas the related investigations in this realm are still lacking to date.^[^ ^33^, ^34^ ^]^

Guided by the earlier considerations, we report herein a reformative current collector for Li metal anode, which is designed by in situ crafting the CuSe granule layer on the dealloyed Cu framework (D‐Cu@CuSe). The dealloyed Cu skeleton that harnesses favorable porosity is beneficial to mitigating volume change and lowering local current density. Meanwhile, CuSe decoration affords sufficient lithiophilic sites to decrease the Li nucleation overpotential as well as homogenize the Li‐ion flux. Overall, such a versatile 3D current collector synergizing the dealloyed porous skeleton and conformal lithiophilic moiety optimizes the Li nucleation behavior and enables a stable high Coulombic efficiency (CE) during repeated Li plating/stripping. Electrochemical tests in combination with theoretical simulations further disclose the Li nucleation mechanism of CuSe moiety. As a result, symmetric cells based on D‐Cu@CuSe‐Li electrode exhibit a low voltage hysteresis and impressive cycling stability even at an elevated DOD of 62.5%, where the dendrite‐free feature during cycling is revealed by operando optical microscopy. More encouragingly, when paired with high‐capacity sulfur cathodes, thus‐assembled Cu@CuSe‐Li||S full cells could sustain a stable cycling performance under a low N/P ratio of 2. Our 3D lithiophilic host design extends the discharge depth and prolongs the cycling lifetime of Li metal anodes, offering a valuable roadmap in the pursuit of pragmatic LMBs under working conditions.

## 2 Results and Discussion

**Figure 1:**
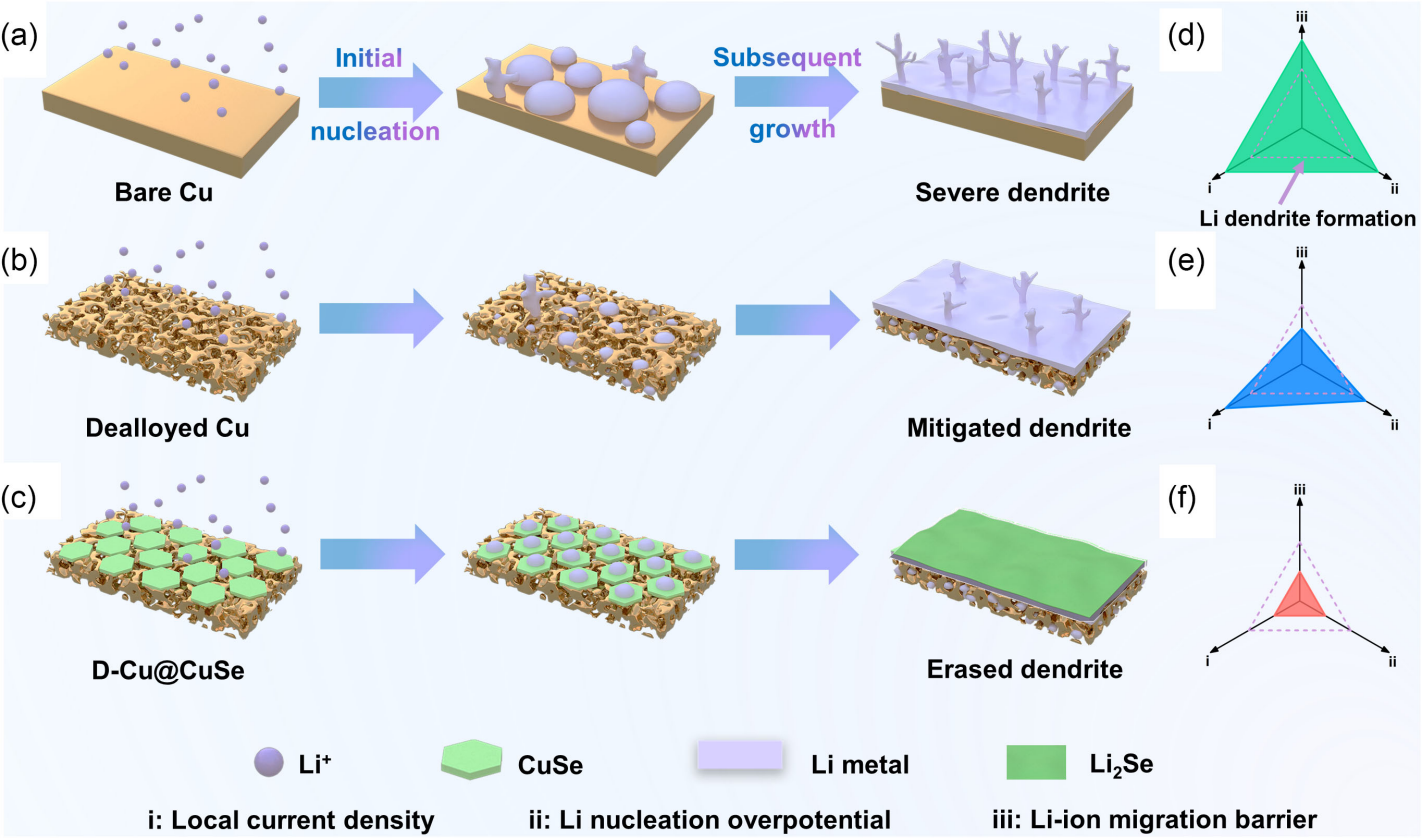
Schematic illustration of the expected Li nucleation behaviors on a) B‐Cu, b) D‐Cu, and c) D‐Cu@CuSe. Synergistic manipulation diagrams of local current density, Li nucleation overpotential, and Li‐ion migration barrier with respect to Li dendrite formation on d) B‐Cu, e) D‐Cu, and f) D‐Cu@CuSe.

Cu foil is recognized as a common current collector for upholding anode materials in rechargeable battery systems. Unfortunately, when loaded with Li metal, thus‐derived Li anode can hardly cushion any volume change and facilitate Li nucleation on account of its 2D and lithiophobic properties, thereby inevitably resulting in severe dendrite formation during repeated Li plating/stripping (**Figure** **1**a). Inspired by the melting point difference between Cu (1083 °C) and Zn (420 °C), D‐Cu skeleton can be readily produced via dealloying the commercial brass plate (incorporating 62% Cu and 38% Zn). The thus‐fabricated 3D current collector is expected to alleviate volume change and reduce local current density, accordingly mitigating the dendritic growth (Figure 1b). Despite these conspicuous advantages, the lithiophobic nature of metallic Cu would still give rise to high Li nucleation overpotential and subsequent dendrite formation. In this sense, lithiophilic CuSe overlayer is further crafted over D‐Cu skeleton to render D‐Cu@CuSe, which will be in situ converted to Li_2_Se as a protective layer, thus further optimizing Li nucleation behavior and enabling a dendrite‐free morphology (Figure 1c). It is proposed that the nucleation and growth behavior of Li on a host is synergistically manipulated by 1) local current density; 2) nucleation potential; and 3) Li‐ion migration barrier. To effectively suppress the dendrite formation (indicated by the violet dashed line in Figure 1d–f), it necessitates the coadjustment of the earlier three parameters to all maintain a reasonably low value. In particular, D‐Cu@CuSe guarantees the concurrent reduction of three parameter values, which is anticipated to inhibit the dendrite formation and ultimately realize a robust Li metal anode.

**Figure 2:**
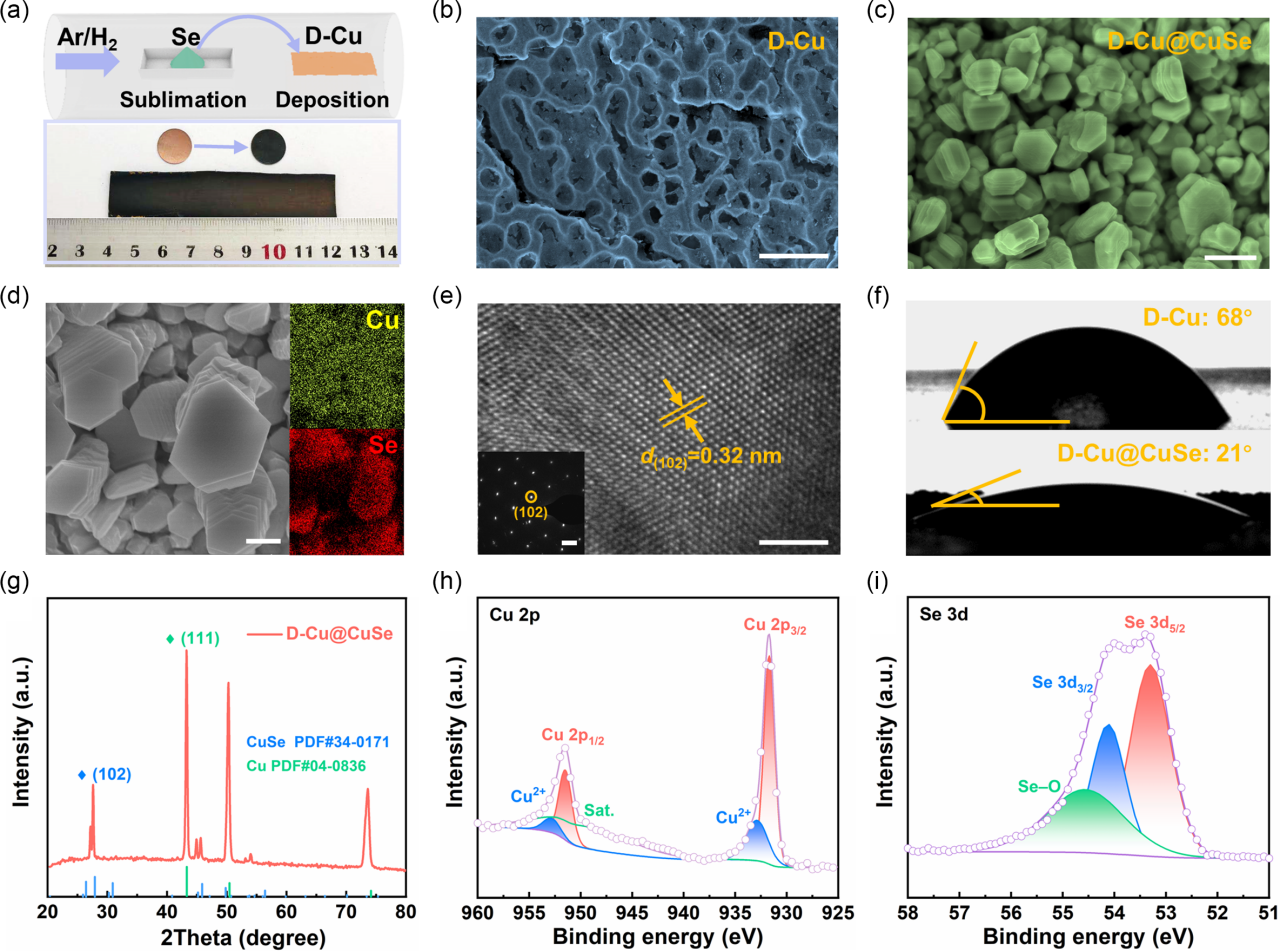
a) Schematic diagram of the synthesis of D‐Cu@CuSe (upper panel) and digital photo of D‐Cu and D‐Cu@CuSe (lower panel). SEM images of b) D‐Cu and c) D‐CuSe. d) High‐magnification SEM image of D‐Cu@CuSe and the corresponding EDS maps. e) HRTEM image of D‐Cu@CuSe. Inset: Corresponding SAED pattern. f) CA measurements of D‐Cu and D‐Cu@CuSe. g) XRD pattern of D‐Cu@CuSe. High‐resolution XPS: h) Cu 2*p* and i) Se 3*d* spectrum of D‐Cu@CuSe. Scar bar: b) 5 μm. c) 2 μm. d) 1 μm. e) 5 nm. Inset: 2 1/nm.

The D‐Cu@CuSe current collector was prepared *via* a sequential two‐step route (**Figure** **2**a; S1, Supporting Information). In the first step, the as‐received brass substrate was subject to a thermal dealloying process under vacuum atmosphere with the aim to remove the Zn element, thereby generating the 3D D‐Cu skeleton with high porosity. In the second step, lithiophilic CuSe granules were in situ crafted on the surface of D‐Cu throughout chemical vapor deposition (CVD). Note that our synthetic strategy is facile and economical, which is envisaged to pursue large‐scale production. The morphological features of products at different synthesis stages were captured by scanning electron microscopy (SEM). As illustrated in Figure 2b and S2 (Supporting Information), D‐Cu exhibits a porous framework architecture with the pore size centered at ≈1 μm, which could offer ample space to accommodate Li metal and cushion the volume change. Figure 2c shows a multitude of hexagonal CuSe plates grown on the surface of D‐Cu skeleton upon a CVD‐mediated selenization process (Figure S3, Supporting Information). The production of CuSe was further confirmed by elemental mapping and transmission electron microscopy (TEM) observation (Figure 2d; S4, Supporting Information). Moreover, high‐resolution TEM (HRTEM) inspection reveals a lattice fringe spacing of 0.32 nm, corresponding to the (102) plane of CuSe (Figure 2e; S5, Supporting Information). The selected‐area electron diffraction (SAED) pattern in Figure 2e (inset) presents clear diffraction spots, which are also indexed as the (102) plane of hexagonal CuSe. To examine the surface wettability of electrolyte for various current collectors, static contact angle (CA) measurement was carried out. As shown in Figure 2f, our fabricated D‐Cu@CuSe displays a markedly smaller CA value (21°) than that of bare Cu (B‐Cu) (58°) and D‐Cu (68°) (Figure S6, Supporting Information). In this sense, the CuSe layer is expected to enable excellent wettability with electrolyte, which is helpful to lower ion‐diffusion resistance at the anode/electrolyte interface.

The X‐ray diffraction (XRD) pattern of D‐Cu@CuSe displays typical signals of Cu (PDF#04‐0836) and CuSe (PDF34#0171).^[^ ^35^ ^]^ It is noted that the dominated peak is assigned to the (102) lattice plane of CuSe (Figure 2g), in good agreement with the HRTEM result. Likewise, the XRD pattern of D‐Cu manifests the diffraction peaks of pure Cu (Figure S7, Supporting Information), indicative of the full removal of Zn species. The Raman spectroscopic study also validates the formation of CuSe overlayer on the surface of D‐Cu, which is evidenced by the characteristic *A*_1g_ peak of CuSe located at 237.8 cm^−1^ (Figure S8, Supporting Information).^[^ ^36^ ^]^ In addition, X‐ray photoelectron spectroscopy (XPS) was used to probe the surface states and chemical environments of D‐Cu@CuSe. As depicted in the Cu 2*p* spectrum in Figure 2h, two contributions appearing at 931.7 and 951.5 eV are related to the signals of Cu^2+^ stemming from the formation of CuSe. As for the Se 3*d* spectrum (Figure 2i), two peaks at 53.3 and 54.1 eV can be assigned to the 3*d*_5/2_ and 3*d*_3/2_ signals of Se^2−^, further suggestive of the successful fabrication of CuSe.

To reflect the conspicuous merits of the D‐Cu@CuSe host for stabilizing Li deposition, a wealth of electrochemical tests were carried out to evaluate its reversibility during repeated Li plating/stripping. CE performance was first examined in the half‐cell configuration assembled by pairing the routine Li metal with D‐Cu@CuSe, while B‐Cu‐ and D‐Cu‐based half cells were also constructed for comparison.^[^ ^37^, ^38^, ^39^ ^]^ It is interesting to note that an apparent discharge platform can be observed at ≈0.8 V (*vs.* Li/Li^+^) during the initial electrochemical activation process (Figure S9, Supporting Information). Correspondingly, the characteristic peaks assigned to Li_2_Se in the XRD pattern further verify the in situ generation of the Li_2_Se protective layer (Figure S10, Supporting Information). The thus‐derived overlayer can improve the stability of the SEI and inhibit dendritic growth.^[^ ^40^ ^]^ More significantly, Li_2_Se is regarded to harness the high ionic conductivity of 10^−5 ^S cm^−1^, which can effectively promote the transport of Li^+^ across the electrode.^[^ ^41^, ^42^ ^]^ In this sense, the in situ‐crafted CuSe nanosheets are beneficial to achieving uniform Li deposition onto D‐Cu@CuSe.

**Figure 3:**
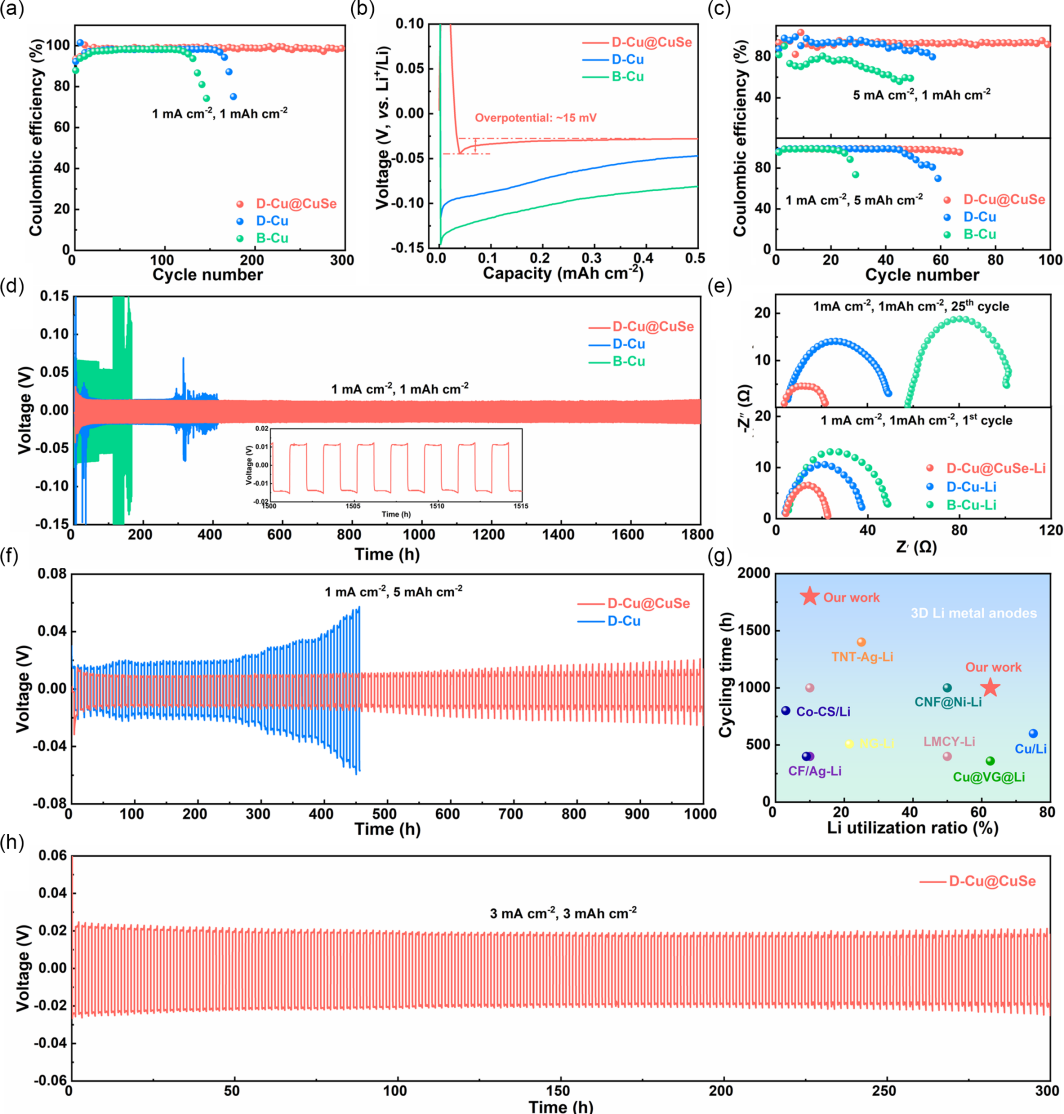
a) CE profiles of B‐Cu, D‐Cu, and D‐Cu@CuSe at 1 mA cm^−2^/1 mAh cm^−2^. b) The voltage−capacity curves at 1 mA cm^−2^/1 mAh cm^−2^. c) CE profiles of B‐Cu, D‐Cu, and D‐Cu@CuSe at 5 mA cm^−2^/1 mAh cm^−2^ and 1 mA cm^−2^/5 mAh cm^−2^. d) Cycling performance of symmetric cells at 1 mA cm^−2^/1 mAh cm^−2^. Inset: Enlarged voltage−time profile of D‐Cu@CuSe‐Li after 1500 h. e) EIS curves of symmetric cells after 1st and 25th cycles. f) Cycling performance of symmetric cells at 1 mA cm^−2^/5 mAh cm^−2^. g) Comparison of cycling time and Li utilization ratio between this work and other start‐of‐the‐art 3D Li metal anode. h) Cyclic performance of D‐Cu@CuSe‐Li‐based symmetric cell at 3 mA cm^−2^/3 mAh cm^−2^.

CE is tightly correlated with Li plating/stripping performance, which can reflect the reversibility and durability of Li metal anode during the electrochemical cycling process. In general, the improvement of CE can readily enhance the cyclic stability of LMBs. As shown in **Figure** **3**a, the CE of D‐Cu@CuSe electrode still is maintained at 98.8% after 300 cycles under a current density of 1 mA cm^−2^ and a plating/stripping capacity of 1 mAh cm^−2^ (denoted as 1 mA cm^−2^/1 mAh cm^−2^), manifesting a highly stable Li plating/stripping behavior. This is in stark contrast to that of B‐Cu and D‐Cu counterparts, both of which are prone to substantially deteriorate after 100 cycles. Figure 3b collects the corresponding voltage−capacity profiles, where the sharp drop pertains to Li nucleation overpotential and the steady voltage platform stands for mass transport‐regulated overpotential. In this regard, the Li nucleation overpotential of D‐Cu@CuSe is estimated to be ≈15 mV, which is significantly lower than that of B‐Cu and D‐Cu, implying that CuSe overlayer harnesses sufficient lithiophilic sites to facilitate Li nucleation. Accordingly, D‐Cu@CuSe readily harvests a high CE and durable cycling performances under conditions regardless of high current density (5 mA cm^−2^/1 mAh cm^−2^) or elevated lithiation capacity (1 mA cm^−2^/5 mAh cm^−2^), far surpassing B‐Cu and D‐Cu electrodes (Figure 3c). In addition, D‐Cu@CuSe also manages to deliver a stable high CE at 2 mA cm^−2^/1 mAh cm^−2^, 3 mA cm^−2^/3 mAh cm^−2^, and 10 mA cm^−2^/1 mAh cm^−2^ (Figure S11, Supporting Information), further corroborating that the lithiophilic CuSe layer aids in stabilizing Li metal anodes.

To elucidate the superiority of D‐Cu@CuSe toward the durable Li metal anode, B‐Cu, D‐Cu, and D‐Cu@CuSe were prestored with 8 mAh cm^−2^ Li metal via an electrodeposition process (Figure S12, Supporting Information). The thus‐derived B‐Cu‐Li, D‐Cu‐Li, and D‐Cu@CuSe‐Li electrodes were subject to symmetric cell testing.^[^ ^43^, ^44^ ^]^ Figure 3d presents the long‐term plating/stripping voltage profiles of B‐Cu‐Li‐, D‐Cu‐Li‐, and D‐Cu@CuSe‐Li‐based symmetric cells at 1 mA cm^−2^/1 mAh cm^−2^. It is observed that D‐Cu@CuSe‐Li implements a stable cyclic performance over 1800 h, whereas B‐Cu‐Li and D‐Cu‐Li exhibit a considerable fluctuation even prior to 400 h cycling. Note that the voltage hysteresis of D‐Cu@CuSe is only 12 mV after 1500 h of cycling (Figure 3d inset), substantiating the effective mitigation of dendritic formation. Figure 3e displays the electrochemical impedance spectroscopy (EIS) curves collected at the 1^st^ and 25^th^ cycle. It is evident that the charge transfer impedance of D‐Cu@CuSe‐Li remains low upon Li plating/stripping, whereas that of B‐Cu‐Li and D‐Cu‐Li significantly augments after electrochemical cycling, demonstrating the excellent cyclic durability of D‐Cu@CuSe‐Li electrode. When the current density reaches 2 and 5 mA cm^−2^, D‐Cu@CuSe‐Li‐based symmetric cells still maintain stable cycling performances (Figure S13 and S14, Supporting Information). Notably, considering the fact that the pragmatic Li metal anode is significantly compromised by shallow DOD, it is of vital importance to evaluate the electrochemical performance under high Li utilization. In this sense, when the lithiation capacity was set at 5 mAh cm^−2^ (corresponding to a high DOD of 62.5%), D‐Cu@CuSe‐Li‐based symmetric cells enable an outstanding cycling operation over 1000 h with a low voltage hysteresis (Figure 3f). In contrast, D‐Cu‐Li exhibits a tremendous voltage fluctuation after 300 h, indicative of an unstable electrode interface caused by abnormal Li depletion and severe dendrite growth. In a broader context, the electrochemical cycling stability of D‐Cu@CuSe‐Li encouragingly outperforms the state‐of‐the‐art 3D Li metal anodes regardless of the DOD value (Figure 3g).^[^ ^19^, ^45^, ^46^, ^47^, ^48^, ^49^, ^50^ ^]^ To showcase the potential application of such a host design, the D‐Cu@CuSe‐Li electrode was further subject to cycling measurements under a stringent working condition of 3 mA cm^−2^/3 mAh cm^−2^. Impressively, it delivers a fairly low and stable voltage hysteresis of ≈20 mV for over 300 h (Figure 3h). In addition, D‐Cu@CuSe‐Li can sustain a stable cycling operation over 30 h at 5 mA cm^−2^/5 mAh cm^−2^ (Figure S15, Supporting Information).

**Figure 4:**
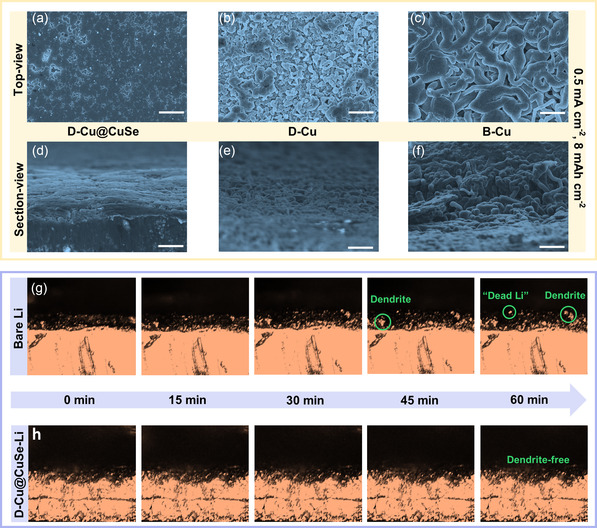
Top‐view SEM images of Li metal deposition on a) B‐Cu, b) D‐Cu, and c) D‐Cu@CuSe at 0.5 mA cm^−2^/8 mAh cm^−2^, respectively. Cross‐sectional SEM images of Li metal deposition on d) B‐Cu, e) D‐Cu, and f) D‐Cu@CuSe at 0.5 mA cm^−2^/8 mAh cm^−2^, respectively. Scale bars in (a‐f): 20 μm. Operando optical microscopy images of Li metal deposition of g) bare Li foil and h) D‐Cu@CuSe‐Li‐based symmetric cells, respectively.

The initial Li deposition morphology is the key to subsequent cyclic durability during repeated Li plating/stripping.^[^ ^51^, ^52^, ^53^ ^]^ **Figure** **4**a–c displays top‐view SEM images of Li deposition morphology on B‐Cu, D‐Cu, and D‐Cu@CuSe current collectors at 0.5 mA cm^−2^/8 mAh cm^−2^, respectively. The corresponding cross‐section SEM views are shown in Figure 4d−f. Apparently, D‐Cu@CuSe exhibits a smooth, compact, and dendrite‐free morphology, manifesting favorable Li nucleation behavior due to abundant lithiophilic sites and low nucleation overpotential. Such a desirable Li deposition morphology is envisaged to enhance the cyclic durability during the long‐term plating/stripping process. In comparison, D‐Cu and B‐Cu display rampant dendrite formation and loose deposition morphology (Figure S16, Supporting Information). In further contexts, real‐time Li plating/stripping dynamics of D‐Cu@CuSe‐Li was monitored by operando optical microscopy measurement at 1 mA cm^−2^ (Figure S17, Supporting Information). As shown in Figure 4g, Li dendrites are prone to grow along the edge of the bare Li foil and become discernible over the course of observation. Suffering from the nonhosting deposition of bare Li foil, a myriad of “dead Li” species, and Li dendrites starts to appear on its surface after 60 min. Nevertheless, 3D D‐Cu@CuSe skeleton enables an ideal Li deposition behavior without the formation of apparent dendrites and “dead Li,” as evidenced in Figure 4h.

**Figure 5:**
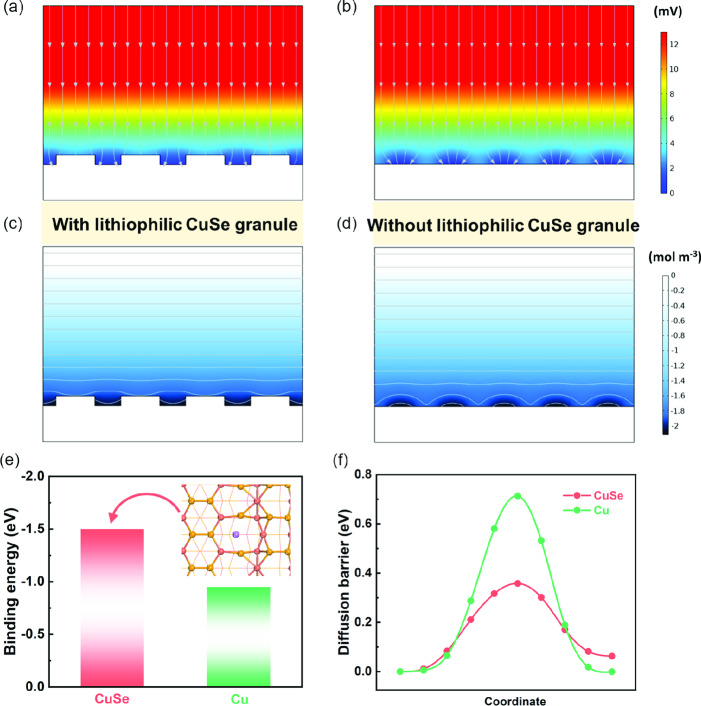
COMSOL Multiphysics simulation of the local current density distribution at the a) D‐Cu@CuSe/electrolyte and b) B‐Cu/electrolyte interface. COMSOL Multiphysics simulation of Li^+^ flux at the c) D‐Cu@CuSe/electrolyte and d) B‐Cu/electrolyte interface. e) Adsorption energies of Li atom on CuSe (102) and Cu (111) plane. Inset: The optimal adsorption model of Li atom on CuSe (102) plane. Red, yellow, and purple ball represents Cu, Se, and Li atom, respectively. f) Migration barriers of Li at CuSe and Cu.

To gain deeper insight into the optimized Li nucleation behavior on D‐Cu@CuSe host, finite‐element method implemented by COMSOL Multiphysics was utilized to probe the local current density and Li‐ion concentration at the interface of anode/electrolyte (Figure S18, Supporting Information).^[^ ^54^, ^55^, ^56^ ^]^ It is demonstrated in **Figure** **5**a that the electric field presents a uniform distribution on the surface of D‐Cu@CuSe, which possibly benefits from the hexagonal CuSe plates. In terms of B‐Cu, current tends to aggregate toward a few tips, leading to an obvious intensity gradient (Figure 5b). Likewise, the Li‐ion concentration displays a more homogeneous distribution on D‐Cu@CuSe as compared with the scenario of B‐Cu, which can be attributed to the abundant Li nucleation sites from lithiophilic CuSe overlay (Figure 5c,d). Overall, our current collector design enables the homogeneous distribution of Li‐ion concentration and electron flux at the interface of D‐Cu@CuSe/electrolyte, which is anticipated to help facilitate Li nucleation and depress dendrite formation.

Theoretical calculations based on density functional theory (DFT) were carried out to further interpret the adsorption and diffusion behavior of Li, thereby deepening the understanding of Li nucleation mechanism under the regulation of CuSe overlayer. CuSe (102) and Cu (111) plane in combination with their possible adsorption sites have been modeled beforehand (Figure S19, S20, Supporting Information). The binding energy of Li atom with the most stable configuration was derived to be −1.50 eV on the CuSe (102) plane, which is apparently lower than that (−0.95 eV) on Cu (111) lattice (Figure 5e). In addition, the bond length between Li and Se on CuSe (102) is close to 2.38 Å, which is shorter than that (2.54 Å) between Li and Cu with respect to Cu (111). These results verify that CuSe owns stronger affinity to Li because of its polar surface induced by the electronegativity diversity between Cu and Se. The migration pathway/barrier of Li on CuSe and Cu support has also been simulated. CuSe possesses a larger 2D diffusion channel with negligible Cu—Se bond breaking during the Li^+^ diffusion process (Figure S21a, Supporting Information). In contrast, Li atom is transported in the edge‐sharing octahedral interval via Cu—Cu bond breaking (Figure S21b, Supporting Information). As depicted in Figure 5f, the migration barrier reaches 0.36 eV on CuSe (102) and 0.71 eV on Cu (111) plane, indicating that Li atom is prone to migrate and redistribute on the surface of CuSe. It is hence concluded that the lithiophilic CuSe layer could not only enhance Li^+^ capture but also accelerate subsequent diffusion kinetics, ultimately optimizing Li nucleation behavior and suppressing Li dendrite growth.

**Figure 6:**
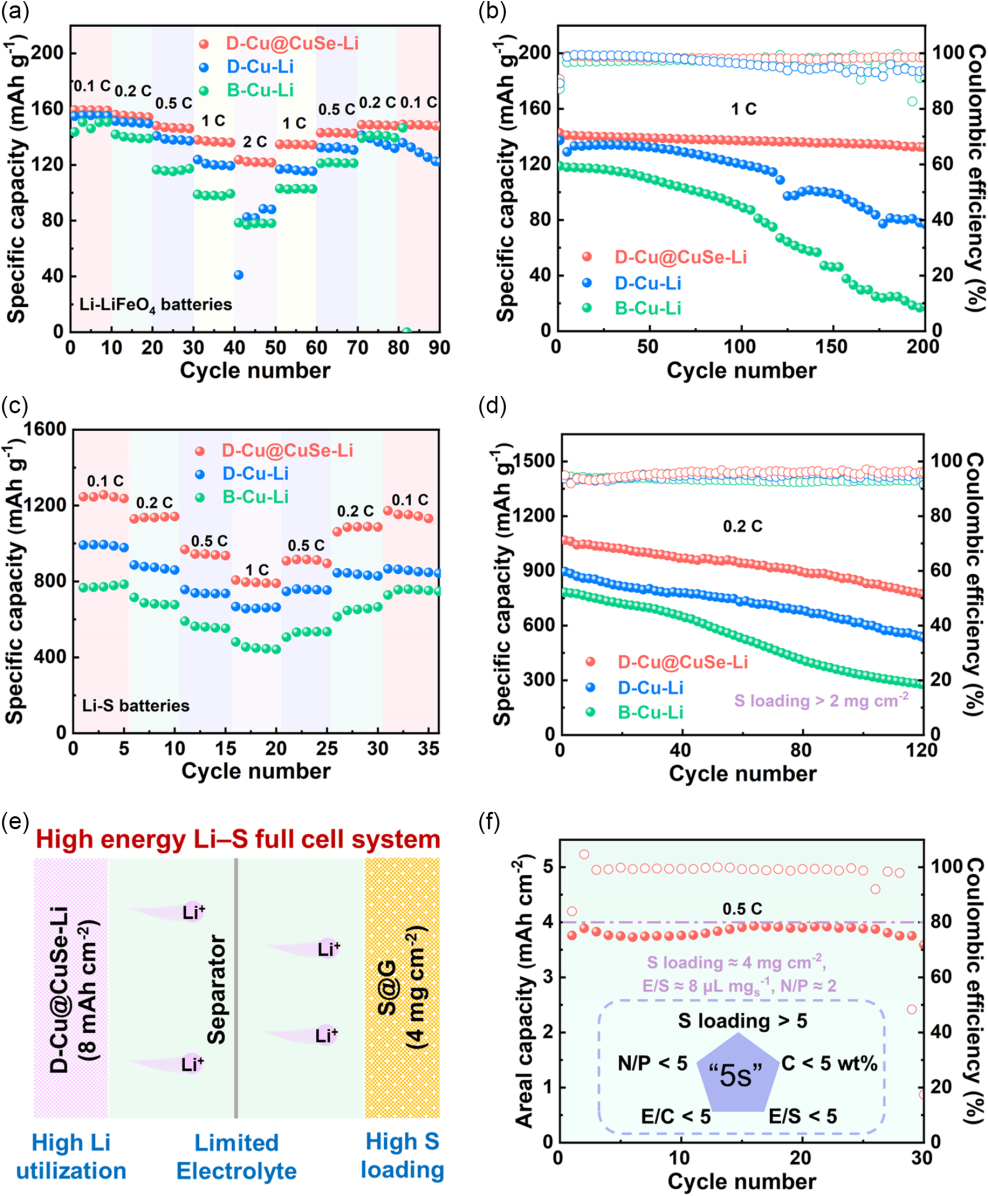
a) Rate performances of B‐Cu‐Li‐, D‐Cu‐Li‐, and D‐Cu@CuSe‐Li‐based Li||LiFePO_4_ full cells. b) Cycling performances at 1 C for 200 cycles. c) Rate performances of B‐Cu‐Li‐, D‐Cu‐Li‐, and D‐Cu@CuSe‐Li‐based Li||S full cells. d) Cycling performances at 0.2 C for 120 cycles. e) Schematic illustration of the construction of the high‐energy Li–S system. f) Cycling performances at 0.5 C for 30 cycles under working conditions. Inset: The “5s” critical metrics required for high‐energy Li–S batteries.

To showcase the prospect of the thus‐designed 3D lithiophilic current collector in the pursuit of the pragmatic Li metal anode, D‐Cu@CuSe‐Li anode was paired with LiFePO_4_ and S cathode to comprehensively evaluate the electrochemical performances of the assembled LMBs (Figure S22–S24, Supporting Information). In terms of the rate performance, D‐Cu@CuSe‐Li||LiFePO_4_ cell exhibits an overwhelming advantage over D‐Cu‐Li||LiFePO_4_ and B‐Cu‐Li||LiFePO_4_, managing to harvest a high initial capacity of 159.4 mAh g^−1^ at 0.1 C and maintain 123.7 mAh g^−1^ at 2 C (**Figure** **6**a). The corresponding galvanostatic charge/discharge (GCD) curves are shown in Figure S25 (Supporting Information), where the reaction polarization of D‐Cu@CuSe‐Li||LiFePO_4_ cell remains at low values under varied current densities. The capacity of its counterparts rapidly falls down to ≈80 mAh g^−1^ at 2 C. When the current density returns to 0.1 C, the capacity value of D‐Cu@CuSe‐Li||LiFePO_4_ shows the best recovery. As for the cyclic performance, D‐Cu@CuSe‐Li||LiFePO_4_ full cell achieves stable cycling at 1 C, accompanied by negligible capacity fading and CE decay over 200 cycles (Figure 6b). In contrast, both the capacity and CE of D‐Cu‐Li||LiFePO_4_ and B‐Cu‐Li||LiFePO_4_ cells suffer from drastic fluctuations during electrochemical cycling, owing to the unstable Li metal anode. Li–S batteries were further assembled to showcase the cyclic durability of D‐Cu@CuSe‐Li anode under stringent conditions pertaining to high DOD. Note that graphene was used as the sulfur host to obtain S@G composite via the thermal melting approach. The XRD pattern indicates the presence of sulfur (PDF#08‐0247), indicating the successful construction of the sulfur cathode (Figure S26, Supporting Information). The D‐Cu@CuSe‐Li||S full cell delivers reversible capacities of 1245.8, 1129.3, 968.5, and 806.6 mAh g^−1^ under varied current densities at 0.1, 0.2, 0.5, and 1.0 C, respectively, far surpassing the D‐Cu‐Li||S and B‐Cu‐Li||S cells (Figure 6c; S27, Supporting Information). Likewise, the cycling performance of the D‐Cu@CuSe‐Li||S cell also outperforms its counterparts to a large extent at 0.2 C (Figure 6d).^[^ ^57^, ^58^, ^59^ ^]^ Notably, D‐Cu@CuSe‐Li||S cell could maintain a stable long‐term operation without a conspicuous plunge over 800 cycles at 1.0 C (Figure S28, Supporting Information). In particular, it is recognized that the concurrent decrease in N/P and electrolyte‐to‐sulfur (E/S) ratios is the key to implementing high‐energy Li–S full batteries (Figure 6e). Manthiram and co‐workers proposed the “5s” critical metrics that are crucial for achieving the high‐energy‐density target of practical Li–S systems (Figure 6f, Inset).^[^ ^60^ ^]^ In this sense, a high‐loading S cathode (≈4 mg cm^−2^), lean electrolyte (8 μL mg_s_^−1^) and a low N/P ratio of ≈2 were used. Encouragingly, the thus‐assembled D‐Cu@CuSe‐Li||S full cell harvests a high areal capacity of ≈4 mAh cm^−2^ and stable cycling performance over 30 cycles at 0.5 C (Figure 6f), holding great promise in pursuing pragmatic LMBs under working conditions.

## 3 Conclusion

In summary, 3D lithiophilic D‐Cu@CuSe skeleton has been developed as a promising current collector to enable dendrite‐free Li deposition and stable operation of Li metal anodes. Symmetric cells based on D‐Cu@CuSe‐Li exhibit low voltage hysteresis and outstanding cyclic stability over 1000 h at a high DOD of 62.5%. The underlying Li nucleation mechanism was unveiled by persuasive electrochemical tests in combination with systematic theoretical analysis. The Cu@CuSe‐Li||S full cell harvests a stable cycling performance under stringent working conditions of extremely low E/S (8 μL mg_s_^−1^) and N/P (≈2) ratio. Our work offers an insight into the design of the practical current collector to simultaneously cushion volume change and restrain dendritic growth in the pursuit of the pragmatic LMBs.

## Conflict of Interest

The authors declare no conflict of interest.

## Supporting information

Supplementary Material

## Data Availability

Research data are not shared.

## References

[1] P. G. Bruce , S. A. Freunberger , L. J. Hardwick , J. M. Tarascon , Nat. Mater. 2012, 11, 19. 10.1038/nmat3191 22169914

[2] J. Xiao , Q. Li , Y. Bi , M. Cai , B. Dunn , T. Glossmann , J. Liu , T. Osaka , R. Sugiura , B. Wu , J. Yang , J. Zhang , M. Whittingham , Nat. Energy 2020, 5, 561.

[3] J. G. Zhang , W. Xu , J. Xiao , X. Cao , J. Liu , Chem. Rev. 2020, 120, 13312. 33174427 10.1021/acs.chemrev.0c00275

[4] Z. Wang , Z. Sun , J. Li , Y. Shi , C. Sun , B. An , H. M. Cheng , F. Li , Chem. Soc. Rev. 2021, 50, 3178. 33480899 10.1039/d0cs01017k

[5] Z. Shi , M. Li , J. Sun , Z. Chen , Adv. Energy Mater. 2021, 11, 2100332.

[6] X. Meng , Y. Sun , M. Yu , Z. Wang , J. Qiu , Small Sci. 2021, 1, 2100021. 10.1002/smsc.202100021 PMC11936033 40213401

[7] X. Gao , Y. Zhou , D. Han , J. Zhou , D. Zhou , W. Tang , J. B. Goodenough , Joule 2020, 4, 1864.

[8] D. Lin , Y. Liu , Y. Cui , Nat. Nanotechnol. 2017, 12, 194. 28265117 10.1038/nnano.2017.16

[9] X. B. Cheng , C. Yan , J. Huang , P. Li , L. Zhu , L. Zhao , Y. Zhang , W. Zhu , S. Yang , Q. Zhang , Energy Storage Mater. 2017, 6, 18.

[10] H. Liu , X. B. Cheng , R. Xu , X. Zhang , C. Yan , J. Huang , Q. Zhang , Adv. Energy Mater. 2019, 9, 1902254.

[11] P. Shi , X. B. Cheng , T. Li , R. Zhang , H. Liu , C. Yan , X. Q. Zhang , J. Q. Huang , Q. Zhang , Adv. Mater. 2019, 31, 1902785. 10.1002/adma.201902785 31379042

[12] Y. Zhu , V. Pande , L. Li , B. Wen , M. S. Pan , D. Wang , Z. F. Ma , V. Viswanathan , Y. M. Chiang , Proc. Natl. Acad. Sci. 2020, 117, 27195. 33060301 10.1073/pnas.2001923117 PMC7959496

[13] Q.-K. Zhang , X.-Q. Zhang , H. Yuan , J.-Q. Huang , Small Sci. 2021, 1, 2100058. 10.1002/smsc.202100058 PMC11935897 40213779

[14] D. Xie , H.-H. Li , W.-Y. Diao , R. Jiang , F.-Y. Tao , H.-Z. Sun , X.-L. Wu , J.-P. Zhang , Energy Storage Mater. 2021, 36, 504.

[15] Z. Zuo , F. He , F. Wang , L. Li , Y. Li , Adv. Mater. 2020, 32, 2004379. 10.1002/adma.202004379 33150673

[16] G. Li , Z. Liu , Q. Huang , Y. Gao , M. Regula , D. Wang , L. Chen , D. Wang , Nat. Energy 2018, 3, 1076.

[17] S. Chi , Q. Wang , B. Han , C. Luo , Y. Jiang , J. Wang , C. Wang , Y. Yu , Y. Deng , Nano Lett. 2020, 20, 2724. 32149520 10.1021/acs.nanolett.0c00352

[18] Z. Li , Q. He , C. Zhou , Y. Li , Z. Liu , X. Hong , X. Xu , Y. Zhao , L. Mai , Energy Storage Mater. 2021, 37, 40.

[19] Z. Hu , Z. Li , Z. Xia , T. Jiang , G. Wang , J. Sun , P. Sun , C. Yan , L. Zhang , Energy Storage Mater. 2019, 22, 29.

[20] J. Pu , J. Li , K. Zhang , T. Zhang , C. Li , H. Ma , J. Zhu , P. V. Braun , J. Lu , H. Zhang , Nat. Commun. 2019, 10, 1896. 31015466 10.1038/s41467-019-09932-1 PMC6478682

[21] J. Chen , J. Zhao , L. Lei , P. Li , J. Chen , Y. Zhang , Y. Wang , Y. Ma , D. Wang , Nano Lett. 2020, 20, 3403. 32239948 10.1021/acs.nanolett.0c00316

[22] Q. Yun , Y. B. He , W. Lv , Y. Zhao , B. Li , F. Kang , Q. H. Yang , Adv. Mater. 2016, 28, 6932. 27219349 10.1002/adma.201601409

[23] L.-M. Wang , X.-K. Ban , Z.-Z. Jin , R.-R. Peng , C.-S. Chen , C.-H. Chen , J. Mater. Chem. A 2021, 9, 13642.

[24] K. Yan , Z. Lu , H. Lee , F. Xiong , P. Hsu , Y. Li , J. Zhao , S. Chu , Y. Cui , Nat. Energy 2016, 1, 16010.

[25] H. Shi , M. Yue , C. J. Zhang , Y. Dong , P. Lu , S. Zheng , H. Huang , J. Chen , P. Wen , Z. Xu , Q. Zheng , X. Li , Y. Yu , Z. S. Wu , ACS Nano 2020, 14, 8678. 32530269 10.1021/acsnano.0c03042

[26] T. Yang , T. Qian , Y. Sun , J. Zhong , F. Rosei , C. Yan , Nano Lett. 2019, 19, 7827. 31577446 10.1021/acs.nanolett.9b02833

[27] P. Zhai , Y. Wei , J. Xiao , W. Liu , J. Zuo , X. Gu , W. Yang , S. Cui , B. Li , S. Yang , Y. Gong , Adv. Energy Mater. 2020, 10, 1903339.

[28] W. Yang , W. Yang , L. Dong , G. Shao , G. Wang , X. Peng , Nano Energy 2021, 80, 105563.

[29] Y. Qiao , H. Yang , Z. Chang , H. Deng , X. Li , H. Zhou , Nat. Energy 2021, 6, 653.

[30] C. Jin , T. Liu , O. W. Sheng , M. Li , T. Liu , Y. Yuan , J. Nai , Z. Ju , W. Zhang , Y. Liu , Y. Wang , Z. Lin , J. Lu , X. Tao , Nat. Energy 2021, 6, 378.

[31] H. Kwon , J. H. Lee , Y. Roh , J. Baek , D. J. Shin , J. K. Yoon , H. J. Ha , J. Y. Kim , H. T. Kim , Nat. Commun. 2021, 12, 5537. 34545077 10.1038/s41467-021-25848-1 PMC8452779

[32] M. Kim , Deepika , S. Lee , M. Kim , J. Ryu , K. Lee , L. Archer , W. Cho , Sci. Adv. 2019, 5, eaax5587. 31692811 10.1126/sciadv.aax5587 PMC6814371

[33] A. Hu , W. Chen , X. Du , Y. Hu , T. Lei , H. Wang , L. Xue , Y. Li , H. Sun , Y. Yan , J. Long , C. Shu , J. Zhu , B. Li , X. Wang , J. Xiong , Energy Environ. Sci. 2021, 14, 4115.

[34] E. Cha , M. D. Patel , J. Park , J. Hwang , V. Prasad , K. Cho , W. Choi , Nat. Nanotechnol. 2018, 13, 337. 29434261 10.1038/s41565-018-0061-y

[35] X. J. Wu , X. Huang , J. Liu , H. Li , J. Yang , B. Li , W. Huang , H. Zhang , Angew. Chem., Int. Ed. 2014, 53, 5083. 10.1002/anie.201311309 24711069

[36] G. B. Sakr , I. S. Yahia , M. Fadel , S. S. Fouad , N. Romčević , J. Alloys Compd. 2010, 507, 557.

[37] C. J. Huang , B. Thirumalraj , H. C. Tao , K. N. Shitaw , H. Sutiono , T. T. Hagos , T. T. Beyene , L. M. Kuo , C. C. Wang , S. H. Wu , W. N. Su , B. J. Hwang , Nat. Commun. 2021, 12, 1452. 33664259 10.1038/s41467-021-21683-6 PMC7933276

[38] P. Zhai , L. Liu , Y. Wei , J. Zuo , Z. Yang , Q. Chen , F. Zhao , X. Zhang , Y. Gong , Nano Lett. 2021, 21, 7715. 34491070 10.1021/acs.nanolett.1c02521

[39] L. Ye , M. Liao , X. Cheng , X. Zhou , Y. Zhao , Y. Yang , C. Tang , H. Sun , Y. Gao , B. Wang , H. Peng , Angew. Chem., Int. Ed. 2021, 60, 17419. 10.1002/anie.202106047 34109719

[40] P. Zhai , T. Wang , H. Jiang , J. Wan , Y. Wei , L. Wang , W. Liu , Q. Chen , W. Yang , Y. Cui , Y. Gong , Adv. Mater. 2021, 33, 2006247. 10.1002/adma.202006247 33630383

[41] F. Liu , L. Wang , Z. Zhang , P. Shi , Y. Feng , Y. Yao , S. Ye , H. Wang , X. Wu , Y. Yu , Adv. Funct. Mater. 2020, 30, 2001607.

[42] Y. Ma , L. Wei , Y. Gu , L. Zhao , Y. Jing , Q. Mu , Y. Su , X. Yuan , Y. Peng , Z. Deng , Nano Lett. 2021, 21, 7354. 34448389 10.1021/acs.nanolett.1c02658

[43] Q. Wu , Y. Zheng , X. Guan , J. Xu , F. Cao , C. Li , Adv. Funct. Mater. 2021, 31, 2101034.

[44] J. Meng , C. Li , Energy Storage Mater. 2021, 37, 466.

[45] Y. Lu , J. Wang , Y. Chen , X. Zheng , H. Yao , S. Mathur , Z. Hong , Adv. Funct. Mater. 2021, 31, 2009605.

[46] S. Huang , L. Chen , T. Wang , J. Hu , Q. Zhang , H. Zhang , C. Nan , L. Fan , Nano Lett. 2020, 21, 791. 33377788 10.1021/acs.nanolett.0c04546

[47] R. Zhang , X. Chen , X. Shen , X. Zhang , X. Chen , X. B. Cheng , C. Yan , C. Zhao , Q. Zhang , Joule 2018, 2, 764.

[48] G. Huang , J. Han , F. Zhang , Z. Wang , H. Kashani , K. Watanabe , M. Chen , Adv. Mater. 2019, 31, 1805334. 10.1002/adma.201805334 30397927

[49] S. Li , Q. Liu , J. Zhou , T. Pan , L. Gao , W. Zhang , L. Fan , Y. Lu , Adv. Funct. Mater. 2019, 29, 1808847.

[50] Y. Gao , H. Hu , J. Chang , Q. Y. Huang , Q. N. Zhuang , P. Li , Z. J. Zheng , Adv. Energy Mater. 2021, 11, 2101809.

[51] G. Li , Z. Liu , D. Wang , X. He , S. Liu , Y. Gao , A. AlZahrani , S. Kim , L. Chen , D. Wang , Adv. Energy Mater. 2019, 9, 1900704.

[52] B. Han , D. Feng , S. Li , Z. Zhang , Y. Zou , M. Gu , H. Meng , C. Wang , K. Xu , Y. Zhao , H. Zeng , C. Wang , Y. Deng , Nano Lett. 2020, 20, 4029. 32343592 10.1021/acs.nanolett.0c01400

[53] Y. Gao , T. Rojas , K. Wang , S. Liu , D. Wang , T. Chen , H. Wang , A. Ngo , D. Wang , Nat. Energy 2020, 5, 534.

[54] C. Li , Z. Sun , T. Yang , L. Yu , N. Wei , Z. Tian , J. Cai , J. Lv , Y. Shao , M. H. Rummeli , J. Sun , Z. Liu , Adv. Mater. 2020, 32, 2003425. 10.1002/adma.202003425 32656930

[55] C. Lu , Z. Gao , B. Liu , Z. Shi , Y. Yi , W. Zhao , W. Guo , Z. Liu , J. Sun , Adv. Funct. Mater. 2021, 31, 2101233.

[56] Z. Hou , Y. Gao , H. Tan , B. Zhang , Nat. Commun. 2021, 12, 3083. 34035276 10.1038/s41467-021-23352-0 PMC8149847

[57] Z. Shi , Z. Sun , J. Cai , Z. Fan , J. Jin , M. Wang , J. Sun , Adv. Funct. Mater. 2021, 31, 2006798.

[58] Z. Shi , Z. Sun , J. Cai , X. Yang , C. Wei , M. Wang , Y. Ding , J. Sun , Adv. Mater. 2021, 33, 2103050. 10.1002/adma.202103050 34463382

[59] Y.-W. Song , Y.-Q. Peng , M. Zhao , Y. Lu , J.-N. Liu , B.-Q. Li , Q. Zhang , Small Sci. 2021, 1, 2100042. 10.1002/smsc.202100042 PMC11935945 40212959

[60] A. Bhargav , J. He , A. Gupta , A. Manthiram , Joule 2020, 4, 285.

