# Supplementary material for: Synergizing Conformal Lithiophilic Granule and Dealloyed Porous Skeleton toward Pragmatic Li Metal Anodes
Source: Small Sci. 2022 Feb 6;2(5):2100110. doi: 10.1002/smsc.202100110 (PMC11935828; doi:10.1002/smsc.202100110)
Supplement: Supplementary file 1 — Supplementary Material [file SMSC-2-2100110-s001.pdf]

Supporting Information

Synergizing Conformal Lithiophilic Granule and Dealloyed Porous Skeleton toward Pragmatic Li Metal Anodes

*Zixiong Shi, Zhongti Sun, Xianzhong Yang, Chen Lu, Shuo Li, Xiaoyu Yu, Yifan Ding, Ting Huang, and Jingyu Sun**

Z. X. Shi, Dr. Z. T. Sun, Dr. X. Z. Yang, Dr. C. Lu, S. Li, X. Y. Yu, Y. F. Ding, Dr. T. Huang, Prof. J. Y. Sun
College of Energy, Soochow Institute for Energy and Materials InnovationS (SIEMIS), Key Laboratory of Advanced Carbon Materials and Wearable Energy Technologies of Jiangsu Province, Soochow University, Suzhou 215006, P. R. China

E-mail: sunjy86@suda.edu.cn

Dr. Z. T. Sun

College of Materials Science and Engineering, Jiangsu University, Zhenjiang 212013, P. R.

China

Keywords: dealloyed, lithiophilic, optimized Li nucleation, high Li utilization, Li metal battery.

Experimental Section

Preparation of D-Cu and D-Cu@CuSe

D-Cu skeleton was obtained *via* a mild annealing process under thermal and vacuum environment. Specifically, commercial brass that incorporates 62% Cu and 38% Zn was subject to a thermal treatment at 600 °C for 1 h. The gas flow was 10 standard cubic centimeters per minute (sccm) Ar and the base pressure was controlled to <10 Pa, thus leading to the removal of Zn element along with formation of porous D-Cu skeleton. The hexagonal CuSe granules were *in situ* grown on the surface of D-Cu *via* an ambient-pressure CVD process. Commercial Se powder was placed at the upstream and the temperature was controlled to 260 ℃D-Cu was placed at the downstream and the temperature was controlled to 600 ℃50 sccm Ar and 50 sccm Ar/H_2 (10% H_2) were employed as the carrier gases. The growth duration was set to 10 min. Finally, the B-Cu, D-Cu, and D-Cu@CuSe were punched into circle discs with a diameter of 13 mm prior to electrochemical use.

Characterizations

Morphology information was collected by Hitachi SU8010 Scanning Electron Microscopy. TEM images and corresponding elemental mappings were captured with the assistance of a Tecnai G2-F20 Transmission Electron Microscopy. XRD patterns were recorded by a Bruker D8 Advance Diffractometer to investigate the crystal structure of samples. XPS spectra were acquired at an Escalab 250Xi spectrophotometer. Raman spectra were obtained on a HORIBA LabRAM HR Evolution Confocal Raman instrument.

Electrochemical tests

All batteries were assembled with standard CR2032 coin-type cells in an Ar-filled glove-box. Celgard 2400 was adopted as the separator and 1 M bis(trifluoromethane sulfonyl) imide (LiTFSI) and 2 wt% $LiNO_3$ additive in 1,3-dioxolane (DOL)/1,2-dimethoxyethane binary solvent (DME) (1:1 by volume) was employed as the electrolyte except for Li||$LiFePO_4$ full battery. With respect to the CE measurement, B-Cu, D-Cu, D-Cu@CuSe and 300-μm thick Li metal foil were employed as the working and counter

electrodes, respectively. The batteries were firstly cycled within a potential range of 0–1 V at 50 μA for five cycles to stabilize the solid electrolyte interphase (SEI). Next, a fixed capacity Li metal was plated on the working electrodes and charged to 0.5 V at a certain current density for each cycle.

B-Cu, D-Cu, and D-Cu@CuSe were plated by Li metal with a prestored capacity of 8 mAh cm^{-2}, thus deriving B-Cu-Li, D-Cu-Li, and D-Cu@CuSe-Li electrodes. The symmetric battery measurement was employed to evaluate the cycling stability and voltage hysteresis of Li metal anode, where the B-Cu-Li, D-Cu-Li, and D-Cu@CuSe-Li were employed as the working electrodes. EIS was examined after 1st cycle and 25th cycle at the frequency ranging from 10^{-2} to 10^{5} Hz.

B-Cu-Li, D-Cu-Li, and D-Cu@CuSe-Li were further used as the anodes in full cell tests (*i.e.*, Li||$LiFePO_4$ and Li||S batteries with limited Li metal). The $LiFePO_4$ cathode was prepared by casting a slurry including 75 wt% $LiFePO_4$, 15 wt% SuperP, and 10 wt% polyvinylidene difluoride (PVDF) onto commercial Al foil with a doctor blading approach. In parallel, S@G composite was prepared according to our previous report[1]. The sulfur cathode was prepared by casting a slurry including 75 wt% S@G, 15 wt% SuperP, and 10 wt% LA133 onto commercial Al foil. Thus-derived materials were punched into circle discs with a diameter of 12 mm and subsequently dried at 60 °C under vacuum atmosphere for 10 h prior to use. As for Li||$LiFePO_4$ battery, an $LiFePO_4$ cathode with an active material loading of ~3.0 mg cm^{-2} was employed as the cathode. The electrolyte was composed of 1 M LiPF6 in ethylene carbonate and diethyl carbonate (1:1 by volume). CV and GCD tests were carried out within a potential range of 2.8–4.0 V. In terms of Li||S battery, an S@G cathode with a S loading of ~1.5 mg cm^{-2} was employed as the cathode. CV and GCD tests were carried out within a potential range of 1.7–2.8 V.

EIS and CV curves were recorded on a CHI 670E electrochemical station. GCD and rate/cycling profiles were collected on a Neware battery testing system.

COMSOL Multiphysics simulations

The COMSOL Multiphysics simulations were performed by Multiphysics 5.5 software based on lithium battery module simulation. The surface diffusion coefficient is 2×10^{-7} m^2 s^{-1}. The Li ion diffusion coefficient in the electrolyte is 2×10^{-9} m^2 s^{-1}, the Li ion concentration is 1×10^3 mol m^{-3}, and the current density is 400 A cm^{-2}.

DFT calculations

All the calculations were performed by spin-polarized density functional theory, using Vienna *Ab-initio* Simulation Package[2] with projector augmented wave pseudopotential[3]. GGA-PBE functional was adopted to deal with electronic exchange-correlation interactions[4]. DFT-D3 correction, schemed by Grimme *et al.*[5], was also considered to accurately compute weak interaction between Li atom and Cu or CuSe system. The kinetic energy cutoff with plane wave basis was set to 400 eV. The convergence criterion for the total energy and residual force per atom was less than 10^{-5} eV and 0.02 eV/Å, respectively. Three layers CuSe (102) lattice plane with periodically repeating (1×2) supercell was constructed, while building four layers Cu (111) surface with (2×2) supercell. Their vacuum layer thickness was 12 Å. The last layer was fixed to bulk position, and other layers and adsorbed Li atom was relaxed fully. The binding energy of Li atom was calculated by the formula: $E_b = E_{total} - E_{surf} - E_{Li}$, E_{total} and E_{surf} are the total energy of CuSe (102) and Cu (111) plane with and without adsorbed Li atom, E_{Li} is the chemical potential of bulk Li. We also computed the migration paths of Li atom in the bulk CuSe with (2×2×1) supercell and Cu system with (2×2×2) supercell using climbing image-nudged elastic band (CI-NEB) method[6].

Supporting Figures

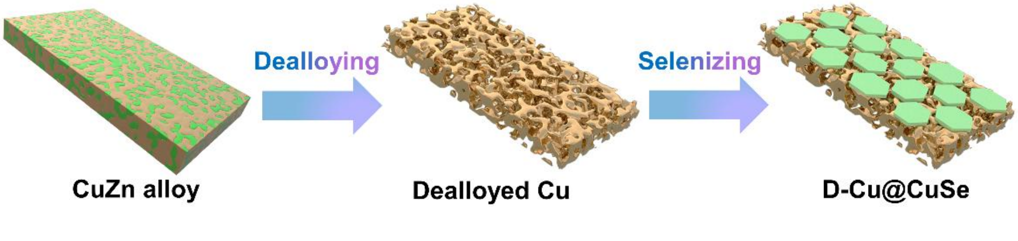

Figure S1. The schematic diagram of sequential two-step synthesis procedures of D-Cu@CuSe.

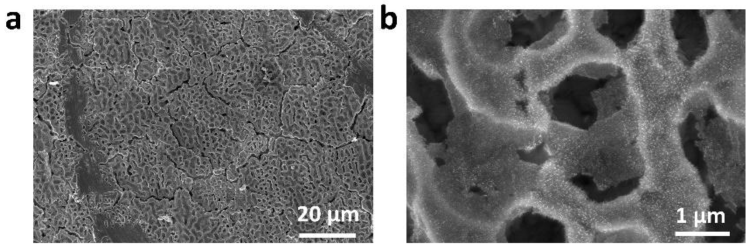

Figure S2. SEM images of D-Cu.

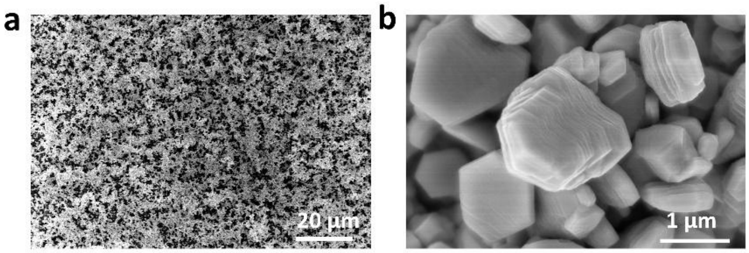

Figure S3. SEM images of D-Cu@CuSe.

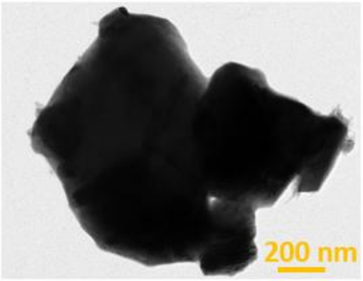

Figure S4. TEM image of D-Cu@CuSe

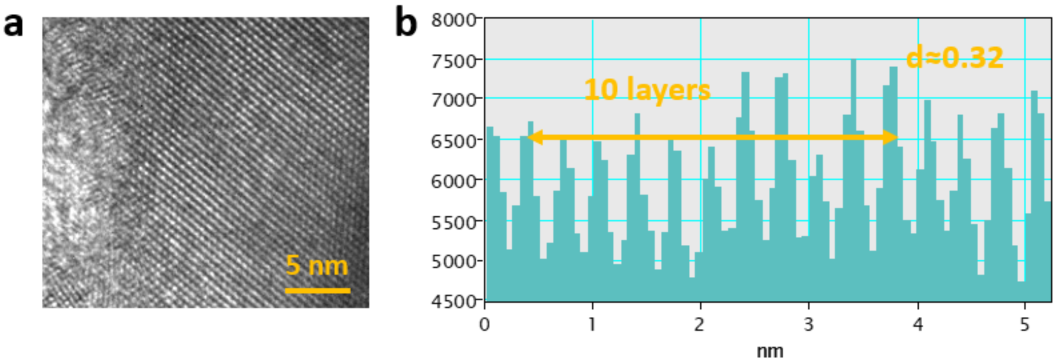

Figure S5. a) HRTEM image of D-Cu@CuSe. b) The corresponding line profiles of the *d*-spacing of CuSe interlayers.

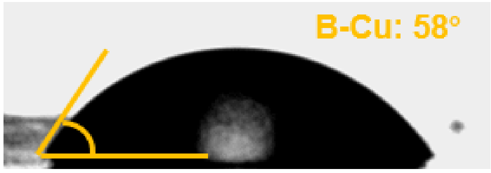

Figure S6. CA measurement of B-Cu.

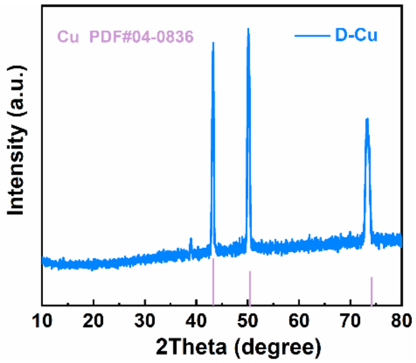

Figure S7. XRD pattern of D-Cu.

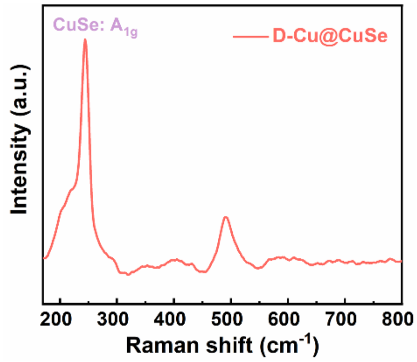

Figure S8. Raman spectrum of D-Cu@CuSe.

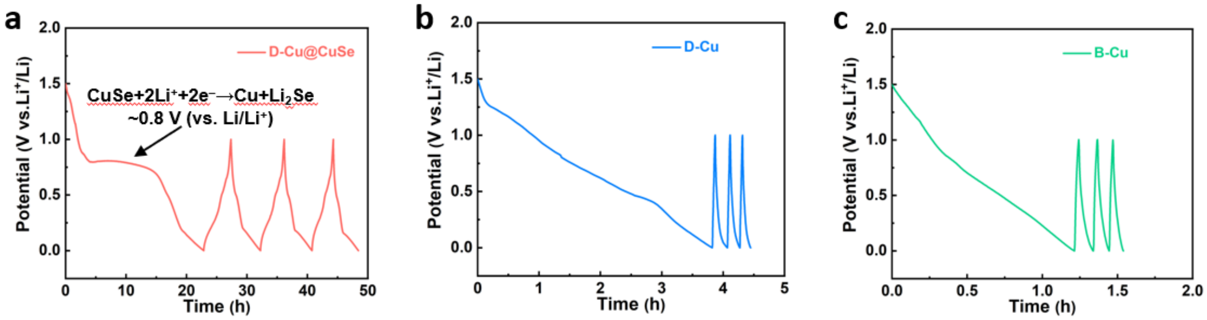

Figure S9. GCD profiles of a) D-Cu@CuSe, b) D-Cu, and c) B-Cu electrodes for three cycles.

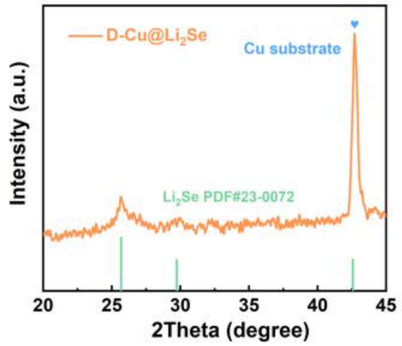

Figure S10. XRD pattern of D-Cu@Li$_2$Se.

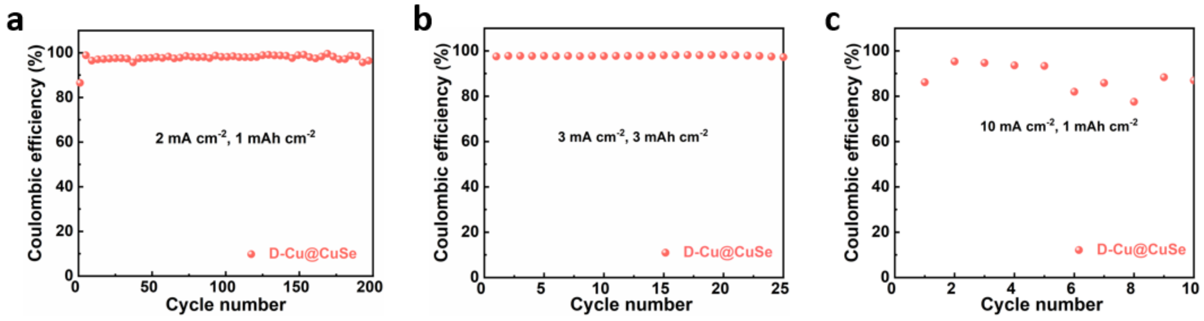

Figure S11. CE of D-Cu@CuSe at a) 2 mA cm^{-2}/1 mAh cm^{-2}, b) 3 mA cm^{-2}/3 mAh cm^{-2}, and c) 10 mA cm^{-2}/1 mAh cm^{-2}.

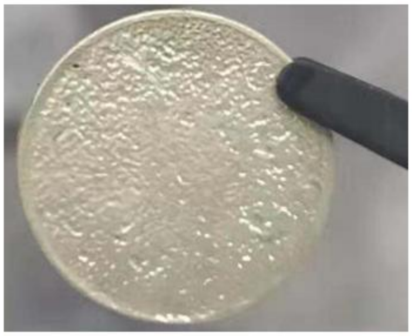

Figure S12. Digital photo of D-Cu@CuSe-Li electrode.

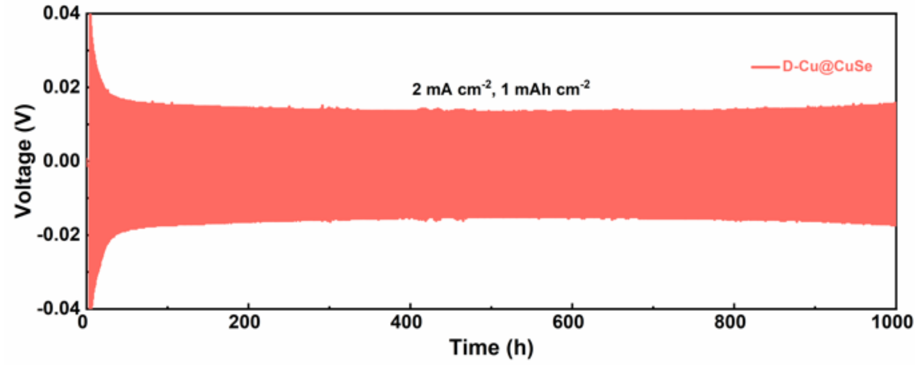

Figure S13. Cycling performance of D-Cu@CuSe-Li based symmetric cell at 2 mA cm^{-2}/1 mAh cm^{-2}.

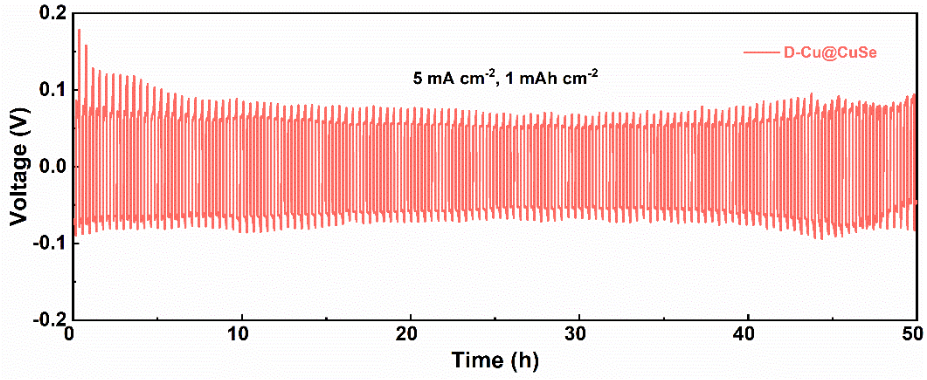

Figure S14. Cycling performance of D-Cu@CuSe-Li based symmetric cell at 5 mA cm^{-2}/1 mAh cm^{-2}.

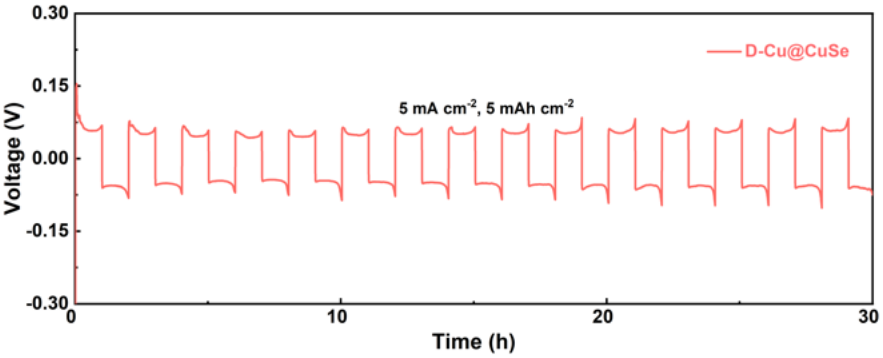

Figure S15. Cycling performance of D-Cu@CuSe-Li based symmetric cell at 5 mA cm^{-2}/5 mAh cm^{-2}.

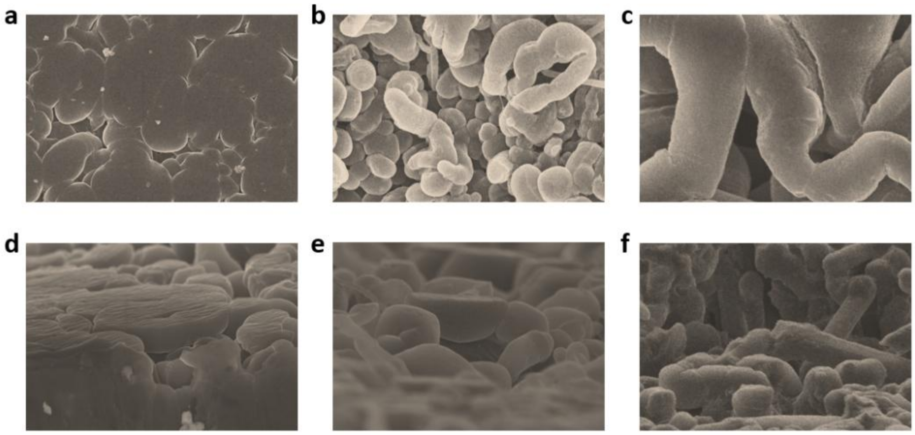

Figure S16. Corresponding enlarged SEM images shown in Figure 4a-f.

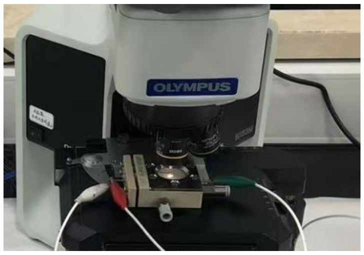

Figure S17. Digital photo of *operando* optical microscopy observation system.

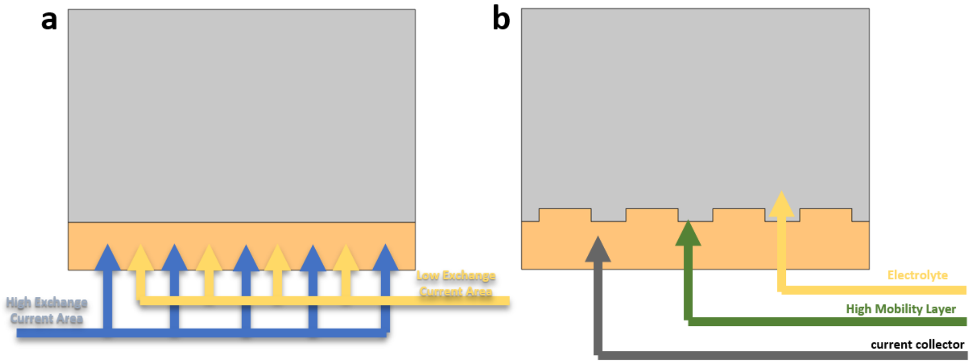

Figure S18. The geometric model of a) B-Cu/electrolyte and b) D-Cu@CuSe/electrolyte in COMSOL Multiphysics simulation.

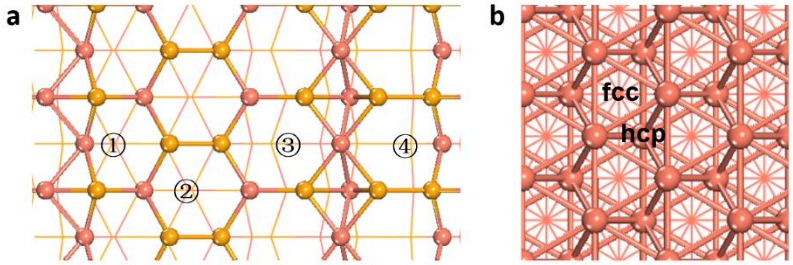

Figure S19. Optimized model of a) CuSe (102) and b) Cu (111) plane. "①, ②, ③, ④" and "fcc, hcp" represent possible adsorption sites of Li atom on the CuSe (102) and Cu (111) surface, respectively. "③" indicates the most stable adsorption configuration of Li atom on CuSe (102) plane. The two adsorption sites of "fcc" and "hcp" own similar binding energy. Red and yellow ball marks Cu and Se atom.

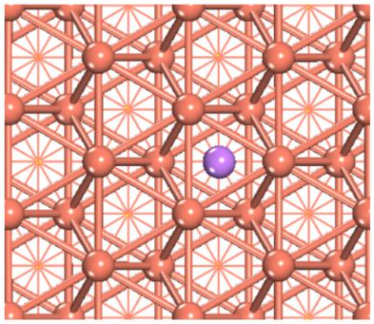

Figure S20. Optimized adsorption model of a Li atom on the Cu (111) plane. Red and purple ball marks Cu and Li atom.

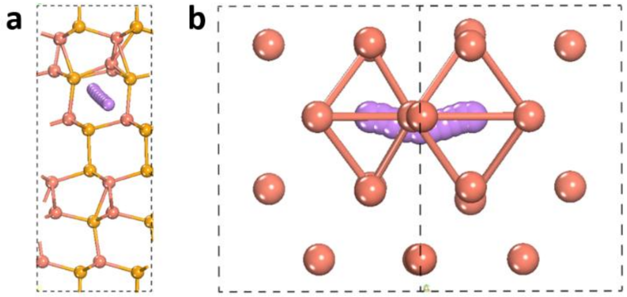

Figure S21. Diffusion pathways of Li atom in the a) CuSe (102) and b) Cu (111) plane. Red, yellow, and purple ball marks Cu, Se, and Li atom, respectively.

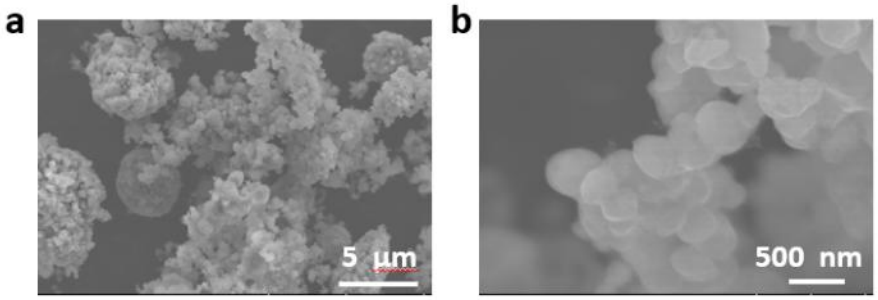

Figure S22. SEM images of $LiFePO_4$.

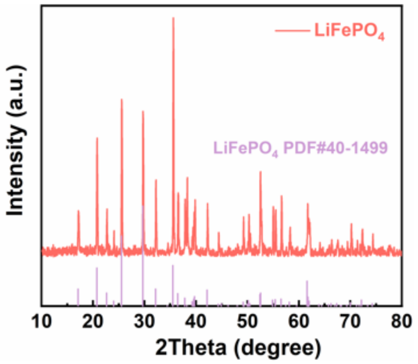

Figure S23. XRD pattern of $LiFePO_4$.

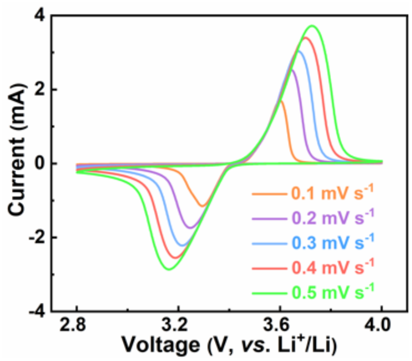

Figure S24. CV curves of $LiFePO_4$ cathode at varied scan rates.

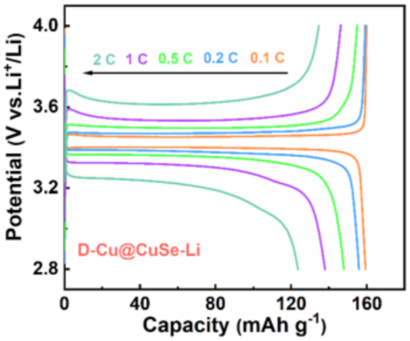

Figure S25. GCD curves of D-Cu@CuSe-Li||$LiFePO_4$ full cell at varied current densities.

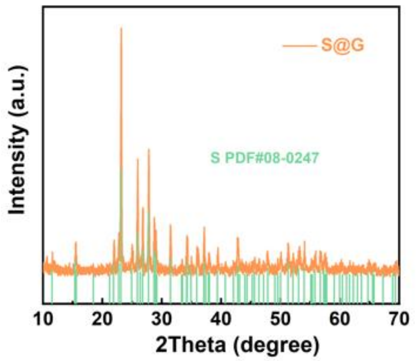

Figure S26. XRD pattern of S@G.

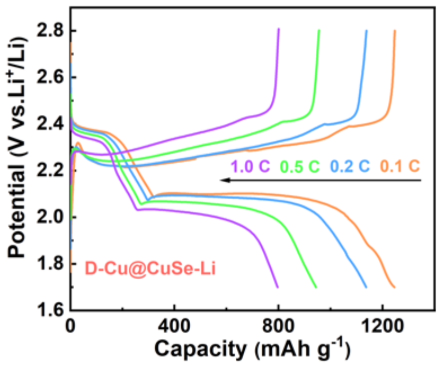

Figure S27. GCD curves of D-Cu@CuSe-Li||S full cell at varied current densities.

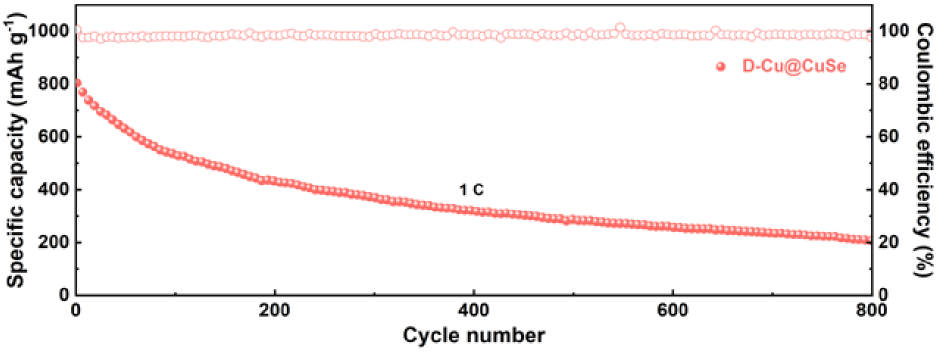

Figure S28. Cycling performance of D-Cu@CuSe-Li||S full cell at 1.0 C for 800 cycles.

Supporting References

[1] Y. Song, W. Zhao, L. Kong, L. Zhang, X. Zhu, Y. Shao, F. Ding, Q. Zhang, J. Sun, Z. Liu, *Energy Environ. Sci.* **2018**, 11, 2620.

[2] G. Kresse, J. Furthmüller, *Comp. Mater. Sci.* **1996**, 6, 15.

[3] P. E. Blöchl, *Phys. Rev. B* **1994**, 50, 17953.

[4] J. P. Perdew, K. Burke, M. Ernzerhof, *Phys. Rev. Lett.* **1996**, 77, 3865.

[5] S. Grimme, J. Antony, S. Ehrlich, H. Krieg, *J. Chem. Phys.* **2010**, 132, 154104.

[6] G. Henkelman, B. P. Uberuaga, H. Jónsson, *J. Chem. Phys.* **2000**, 113, 9901.
